# OptimEYEzing Emergency Skills: A Novel Model for Ocular Procedural Education for Emergency Medicine Residents

**DOI:** 10.5070/M5.52212

**Published:** 2026-04-30

**Authors:** Carrie Maupin, Ambika Anand, Grace Hickam, Stephen Miller

**Affiliations:** * Virginia Commonwealth Univrsity School of Medicine, Department of Emergency Medicine, Richmond, VA

## Abstract

**Audience:**

This model for ocular procedural education is designed to instruct emergency medicine residents of all levels of training.

**Introduction:**

Ocular complaints are a common presentation to the emergency department (ED) with some studies quoting as many as two to three million ocular-related visits annually.^1^,^2^ These complaints can range from minor issues, such as corneal abrasions, to more serious conditions that require prompt evaluation and management, such as retrobulbar hematomas. Emergency medicine physicians are often the first-line providers assessing these complaints, so it is imperative that they feel adept in recognizing and managing these complaints.^2^

Despite the frequency of ocular-related visits, ophthalmologic training among emergency medicine residents is often limited. Studies have reported that residents receive less than ten hours of formal ophthalmology training throughout their residency.^2^,^3^ One study revealed that many emergency medicine physicians in the United States are not confident in using basic ophthalmic tools, conducting eye exams, diagnosing ophthalmic complaints, or performing vision-saving procedures.^2^ This lack of formal training makes it difficult for residents to confidently diagnose and manage ocular conditions in the emergency department, both during residency and in their future practice. On review of the literature, there have been developments of procedural models for the practice of ophthalmology skills including removal of corneal foreign bodies utilizing swine eyes, tonometry using water balloons, and lateral canthotomy using a cadaver eye.^4^.^5^ However, cadavers can be very difficult to obtain, and expensive and swine eyes are not reusable or easy to store.

There is a need for cost-effective, hands-on, ocular training models to help bridge this learning gap and increase residents’ comfort with common ocular complaints and procedures – including foreign body removal, lateral canthotomy, fluorescein staining, and intraocular pressure (IOP) measurements. Integrating hands-on ocular training models into medical education can ultimately lead to better patient outcomes.

**Educational Objectives:**

By the end of this session, learners will be able to: 1) identify signs and symptoms of ocular emergencies, 2) appraise for indications to perform ocular procedures, 3) demonstrate procedural competence in ocular foreign body removal, fluorescein staining, lateral canthotomy, and intraocular pressure (IOP) measurements, 4) relate increased procedural confidence with ocular procedures.

**Educational Methods:**

We developed two gelatin-based eye models that are low-cost and can be easily replicated. The first was created with Knox^®^ gelatin which can be easily made at home. The second was made with Humimic Medical™ synthetic gelatin which can be easily melted down and re-used. The gelatin base mimics the eye and allows for practice of foreign body removal techniques. Different concentrations of gelatin can be used to simulate different IOPs to practice IOP measurement. Fluorescein stain can be applied to the gelatin models to mimic corneal abrasions/ulcerations with use of a Woods lamp. Finally, the gelatin eye models can be placed into an existing 3D printed face model for lateral canthotomy procedural practice, utilizing rubber bands as the ligaments.^6^ Our institution provides access to a 3D printer for both students and faculty. The average total time to create all material for this ocular session was about 20 hours, though many elements of this model can be utilized multiple times. Utilizing low-cost material, the total cost of one reusable model is about 30 United States Dollars (USD).

**Research Methods:**

Twenty-one residents, ranging from their first year of training to their third year of training, participated in the session. A pre-survey was administered to all participants (supplemental materials). The pre-survey was broken down into each ocular procedure listed above, and the participants were asked if 1) they had ever performed the procedure, 2) if they had ever consulted ophthalmology for assistance with the procedure, and 3) to rate their comfort level with the procedure using a Likert scale (1=not at all comfortable to 5=very comfortable). After completion of the session, a post-survey (supplemental materials) was administered to participants, again broken down by each ocular procedure, asking 1) to rate their comfort level with the procedure using the same Likert scale and 2) if they would plan to consult ophthalmology for assistance with the procedure in the future.

Variables were summarized using percentages and frequencies for categorical variables, and means and ranges for continuous variables were presented. Using an α-value of 0.05, a T-test for independent samples was performed to determine if a difference between comfort levels before and after each activity exists.

**Results:**

Twenty-one participants took part in the learning session; all participants were emergency medicine residents, ranging from post-graduate year (PGY)1s to PGY-3s. Of the participants, 100% had performed fluorescein staining, 24% had performed ocular foreign body removal, 95% had performed IOP measurements utilizing a Tono-Pen,^®^ and 14% had performed a lateral canthotomy (all in conjunction with ophthalmology) prior to this activity. There was a statistically significant increase in self-reported comfort level with each of these procedures after the activity: for fluorescein staining, comfort level increased from 4.1 to 4.6 (p-value 0.04); for ocular foreign body removal, comfort level increased from 2.3 to 3.9 (p-value <0.01); for IOP measurements utilizing a Tono-Pen,^®^ comfort level increased from 4.1 to 4.8 (p-value 0.01); for lateral canthotomy, comfort level increased from 1.7 to 3.5 (p-value <0.01). Please see Table 3 for details. Resident comments included: “Great simulations,” “Helpful, want slit lamp,” “Amazing!” and “Great sims!”

**Discussion:**

Feedback from residents was favorable, and their comfort level with each of these ocular procedures improved after the activity. We believe this tool can offer simulation of these procedures at a relatively low cost with reusable materials to improve both comfort level and procedural competence in emergency medicine residents.

**Topics:**

Ocular procedures, lateral canthotomy, Tono-Pen,^®^ IOP measurement, ocular foreign body removal, fluorescein staining, Wood’s lamp.

## USER GUIDE

**Table:** 

List of Resources:	
Abstract	21
User Guide	24
Appendix A: PowerPoint Presentation	29
Appendix B: Pre-Survey	30
Appendix C: Post-Survey	32

**Learner Audience:**

Senior Medical Students, Interns, Junior Residents, Senior Residents, EM Attendings, EM APCs

**Time Required for Implementation:**

Learning sessions should take approximately 60 minutes: 15 minutes for the introductory PowerPoint to discuss each procedure and 45 minutes for procedure practice and debrief (20 minutes for the lateral canthotomy station, 10 minutes at each other station, 5 minutes for debrief). The instructor will spend approximately 20 hours creating this innovation.

**Recommended Number of Learners per Instructor:**

We recommend 3–4 learners per instructor.

**Topics:**

Ocular procedures, lateral canthotomy, Tono-Pen,^®^ IOP measurement, ocular foreign body removal, fluorescein staining, Wood’s lamp.

**Objectives:**

By the end of this session, learners will be able to:

1. Identify signs and symptoms of ocular emergencies
2. Appraise for indications to perform ocular procedures
3. Demonstrate procedural competence in ocular foreign body removal, fluorescein staining, lateral canthotomy, and intraocular pressure (IOP) measurements
4. Relate increased procedural confidence with ocular procedures.

### Linked objectives and methods

The selected format and methods are designed to provide hands-on, experiential learning, which aligns with the goal of building confidence and competence in recognition of ocular emergencies and procedural performance of ocular foreign body removal, fluorescein staining, lateral canthotomy, and intraocular pressure (IOP) measurements. By dividing the session into three distinct stations, each focused on a specific skill, learners can engage in focused practice that mimics real-world scenarios. The use of 3D printed models and gelatin eyeballs enhances realism and allows for repetitive practice which is critical for skill acquisition. The introductory PowerPoint and video instructions provide foundational knowledge and a visual guide, ensuring that learners understand the procedures before practicing them.

The conceptual framework guiding this content development is based on Kolb’s Experiential Learning Theory and constructivism, which underscore the importance of concrete experience and active experimentation in adult learning.^1^ By rotating through the stations, learners can immediately apply the theoretical knowledge gained from the introductory lecture, thereby reinforcing their learning and improving retention.

### Recommended pre-reading for instructor

- Provided “Ocular Emergencies” PowerPoint presentation (in supplemental materials)
- Mason J. *Lateral Canthotomy*. EM:RAP.ORG; 2016. Accessed March 21, 2024. https://www.emrap.org/hd/episode/lateral/lateral
- Babineau MR, Sanchez LD. Ophthalmologic procedures in the emergency department. *Emerg Med Clin North Am*. 2008;26(1):17-vi. doi:10.1016/j.emc.2007.11.003

### Learner responsible content (LRC)

None; all material will be reviewed during the activity.

### Session Implementation

To prepare for the session, instructors should set up three stations: 1) lateral canthotomy, 2) IOP measurement, and 3) ocular foreign body removal and fluorescein staining. The lateral canthotomy station should include the 3D printed mask (pre-loaded with rubber bands and with 3M^TM^Coban^TM^ wrap in place), hemostat, scissors, forceps, and gelatin eyeball. The IOP measurement station should include the Tono-Pen^®^ and several gelatin eyeballs (of differing concentrations to mimic different pressures). The ocular foreign body removal and fluorescein staining station should include the Wood’s lamp, fluorescein stain, gelatin eyeballs (with the silicon mold), saline syringes, 3–5 cc syringe, 25–30-gauge needle, and foreign bodies (such as small rocks/dirt). The foreign bodies should be preloaded into the gelatin eyeballs.

The session begins with an introductory PowerPoint discussing each ocular procedure along with a video instruction on how to perform a lateral canthotomy (found in supplemental materials).

### List of items required to replicate this innovation

1. 25–30-gauge needle
2. 3D-printed mask
  ○ Lulzbot® 3D printer with PLA filament
3. 3D-printed rubber band marker
4. 3M^TM^ Coban^TM^
5. Fluorescein stain
6. Forceps
7. Foreign bodies (rocks/dirt)
8. Gelatin eyes
  ○ Humimic Medical™ gel
  ○ Knox® gelatin
  ○ Silicon mold
9. Hemostat
10. Iris/suture scissors
11. Lidocaine (or saline bottle labeled as lidocaine)
12. Normal saline flush
13. Rubber bands (3.5 in × 0.5 in, #84 elastic bands)
14. Syringe
15. Tono-Pen®
16. Woods Lamp

### Approximate cost of items to create this innovation

Assuming access to a 3D printer, the mask and marker cost $2.86 USD to create with PLA filament (total of 130g of filament used). Knox^®^ gelatin eyeballs cost $2 USD for total of ~15–20 eyeball models. Humimic Medical™ gel eyeball models cost $16 USD for a total of ~14 reusable models (models can be melted and reformed). The silicone mold used to form the eyeball models is $5 USD and is reusable. Total cost of a single model is approximately $30 USD. For total material costs, please see Table 1. Other materials required that should be available through the ED stock include: 3M^TM^Coban,^TM^ hemostat, scissors, forceps, Tono-Pen,^®^ Wood’s lamp, fluorescein stain, saline syringes, 3–5cc syringe, 25–30g needle, Lidocaine (or saline bottle labeled lidocaine if unable to access). If these are not readily available, cost for these materials (excluding the Woods Lamp and Tono-Pen^®^) is approximately $90 (though includes bulk buying of some materials). Foreign bodies, such as rocks/dirt, are also required.

### Detailed methods to construct this innovation

#### Gelatin Preparation

1. Choose gelatin consistency
  - *Softest*: 1 oz gelatin (4 packets) with 1.75 cups of water (IOP 15–30).
  - *Middle*: 1 oz gelatin (4 packets) with 1.5 cups of water (IOP 30–50).
  - *Firmest*: 1 oz gelatin (4 packets) with 1.25 cups of water (not suitable for accurate IOP).
2. Combine gelatin powder with the chosen amount of cold water in a saucepan. Let it sit for 1 minute, and then apply medium heat while stirring until fully dissolved.
3. Lightly oil the mold with cooking spray to facilitate removal. Pour the gelatin mixture into the mold and place it on a flat plate or baking sheet. Refrigerate for at least 8 hours, preferably overnight. Use the gelatin within 2 days to avoid mold growth and dispose of gelatin after use.

#### Humimic Medical™ Gel Preparation

1. Preheat oven to 250°F.
2. Place the Humimic Medical™ gel in an oven-safe loaf pan and heat until fully melted (15–30 minutes).
3. Carefully pour the hot gel into molds (can use liquid measuring cup). Allow it to cool for at least 6 hours, preferably 24 hours.
4. After use, gently rinse with water. The gel can be re-melted but note that fluorescein will permanently stain it.

#### Lateral Canthotomy Model Creation

**Figure 1:**
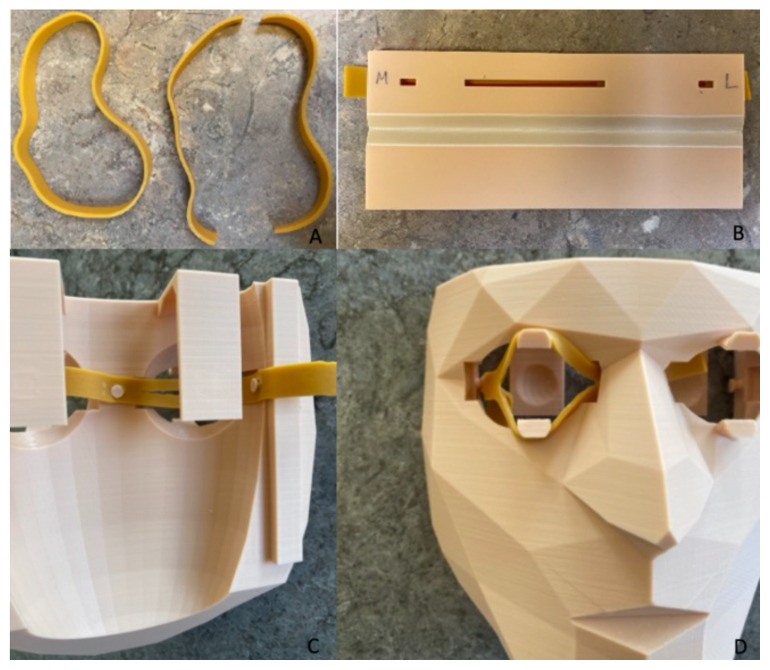
Creation of the lateral canthotomy mask trainer. A. Cut the rubber bands in half. B. Mark rubber bands with 3D printed marker. C. Attach rubber bands to the inner side of mask. D. Attach rubber bands to the outer side of the mask.

**Figure 2:**
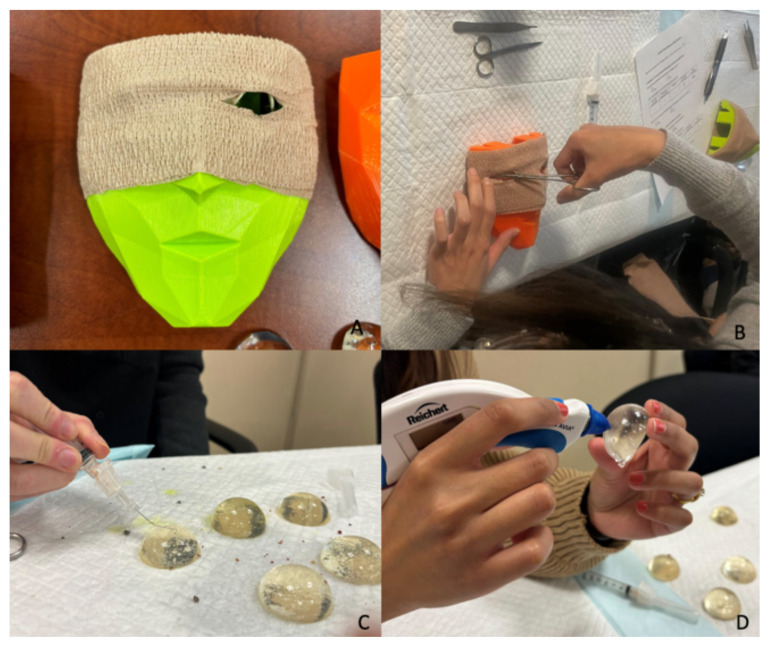
Layout of each procedure station. A. Lateral canthotomy 3D printed mask with 3M^TM^Coban^TM^ in place. B. Resident utilizing the lateral canthotomy trainer. C. Resident performing ocular foreign body removal. D. Resident performing IOP measurement.

1. Print the 3D mask and marker tool utilizing the 3D lateral canthotomy file^7^ and the specifications outlined in Table 2 (assuming use of a Lulzbot^®^ 3D printer).
2. Use 3.in x 0.5in rubber bands (#84 elastic bands), cut in half. Use the 3D printed marker tool to mark and cut the rubber bands as per instructions (see Figure 1).
3. Place rubber bands in the 3D printed mask (see Figure 1).
  - Wrap the mask in 3M^TM^Coban.^TM^ Cut slits into the 3M^TM^Coban^TM^ over the eye openings and insert the gelatin or Humimic Medical™ gel eyeball into the model (see Figure 2).

### Results

Twenty-one emergency medicine residents participated in the above-described curriculum and completed the pre- and post-surveys. The participants included seven PGY-1s, five PGY-2s, and nine PGY-3s.

Results of the pre-survey showed that 100% of the participants had performed fluorescein staining, 24% had performed ocular foreign body removal, and 95% had performed IOP measurements utilizing a Tono-Pen®. Fourteen percent of participants reported in the pre-survey that they had performed a lateral canthotomy, but all instances were in conjunction with ophthalmology. The average comfort level of residents, on a 5-point Likert scale, for each procedure was as follows: fluorescein staining, 4.1; ocular foreign body removal, 2.3; IOP measurement, 4.1; and lateral canthotomy, 1.7. For all procedures, the lowest mean comfort level was found in the PGY-1 group (Table 3).

There was a statistically significant increase in self-reported comfort level with each of these procedures after the activity: for fluorescein staining, comfort level increased from 4.1 to 4.6 (p-value 0.04); for ocular foreign body removal, comfort level increased from 2.3 to 3.9 (p-value <0.01); for IOP measurements utilizing a Tono-Pen®, comfort level increased from 4.1 to 4.8 (p-value 0.01); for lateral canthotomy, comfort level increased from 1.7 to 3.5 (p-value <0.01). Among training levels, this statistically significant increase in comfort level persisted for both foreign body removal and lateral canthotomy. Please see Table 3 for details. Resident comments included: “Great simulations,” “Helpful, want slit lamp,” “Amazing!” and “Great sims!”

Limitations of the review of this activity include this being a single-center study with a small total number of participants. Another limitation of the activity is the orientation of the eye in the foreign body removal. Typically, foreign body removal is performed with the patient sitting upright, but during this activity the gelatin eye is flat on the table as if the patient were lying flat. However, the participants did enjoy the activity, and educational objectives appear to have been met based on the feedback received.

### Tips for successful implementation

#### Intraocular Pressure (IOP) Measurement Setup

1. Choose models with different gelatin concentrations to simulate varying IOP levels.
2. Gently hold the eyeball model between thumb and index fingers on the edges, with the flat side facing away from the learner, in the air so it is not in contact with the table (see Figure 2).
3. Use the TonoPen^®^ as per manufacturer instructions to measure pressure.

#### Ocular Foreign Body Removal & Fluorescein Staining Setup

1. Place rocks or dirt into the gelatin surface to simulate a defect.
2. Place the gelatin eyeball on the table, flat side down. Participants will use the needle (25 to 30-gauge) and syringe to remove the foreign body tangentially with the bevel facing up (see Figure 2).
3. Participants will apply fluorescein with saline and examine the defect using the Wood’s lamp.

#### Lateral Canthotomy

1. Once the finished model is in place, participants will simulate injecting lidocaine, clamp with hemostats, cut the “skin” (3M^TM^Coban^TM^ ) with scissors, and cut the inferior and superior ligaments (rubber bands) (see Figure 2).

### Associated content

- “Ocular Emergencies” PowerPoint presentation
- 3D Lateral Canthotomy Mask File^7^
- PowerPoint Presentation
- Pre-Survey
- Post-Survey

## Supplementary Information



## Figures and Tables

**Table 1:** Model material & costs

Material	Price

25–30g needle	$9.79 (100 pack, 25g needle) https://a.co/d/gRJ7AvR

3D printed lateral canthotomy mask (reusable) Lulzbot® 3D printer with PLA filament	$2.96 (total of 130 gauge of PLA filament used)
	2,995 (price not included, assuming institutional access to 3D printing) https://tinyurl.com/mruwczwb
	3D lateral canthotomy mask^7^ file: https://tinyurl.com/3j756pjd

3D-printed rubber band marker	Included in pricing for mask above

3MTMCobanTM	$5.74 https://a.co/d/h6kFrgn

Fluorescein stain	$19 (50 pack) https://a.co/d/eXbGbIo

Forceps	$11.99 https://a.co/d/eAedMgu

Foreign bodies (rocks/dirt)	$0

Gelatin eyes	
Humimic Medical™ gel (reusable)	$36 (per pound)
Knox® gelatin	$16 (32 packets)
Silicone molds (reusable)	$9.99 (2 sets of molds)

Hemostat	$7.99 https://a.co/d/fGlPmQG

Iris/suture scissors	$9.99 (pack of 3) https://a.co/d/8Z4z1Uj

Lidocaine (or saline bottle labeled as lidocaine)	$8.38 https://tinyurl.com/38kwjbw8

Normal saline flush	$1.15 https://tinyurl.com/5efnaxxy

Rubber bands (3.5in x 0.5in, #84 elastic bands)	$7.99 (pack of 50)

Syringe	$18.99 (pack of 100) https://a.co/d/4pXm5tC

Tono-Pen,®	$5,490 (price not included, assuming institutional access) https://tinyurl.com/yxa7beyk

Woods Lamp	$569.25 (price not included, assuming institutional access) https://tinyurl.com/mwuvbuye

**Table 2:** 3D printing specifications used to create the lateral canthotomy mask & marker tool

**Printer**	Lulzbot^®^ 3D Printer
**Mask File**	https://tinyurl.com/3j756pjd
**Material**	PolyMaker PolyLite^TM^ PLA filament with a 2.85 mm diameter
**Scale**	95%
**Layer height**	0.3 mm
**Shell**	1 mm wall thickness with wall count 2
**Infill**	15%
**Support**	Zig zag
**Plate adhesion**	Skirt adhesion
**Total time**	8 hr 45 min

**Table 3:** Results by training level and procedure type

Procedure	Training Level	Number (%) who have performed the procedure	Average comfort level* before activity (range)	Average comfort level* after activity (range)	T-test (p-value <0.05)
Fluorescein staining	PGY1 (N=7)	7 (100%)	3.7 (2–5)	4.5 (4–5)	0.07
	PGY2 (N=5)	5 (100%)	3.8 (3–5)	4.4 (3–5)	0.37
	PGY3 (N=9)	9 (100%)	4.6 (4–5)	4.8 (4–5)	0.43
	**Total** (N=21)	21 (100%)	4.1 (2–5)	4.6 (3–5)	0.04
Ocular foreign body removal	PGY1 (N=7)	1 (14%)	2.1 (1–4)	3.9 (3–5)	<0.01
	PGY2 (N=5)	1 (20%)	2.2 (1–4)	3.8 (3–4)	0.03
	PGY3 (N=9)	3 (33%)	2.4 (1–4)	4 (3–5)	<0.01
	**Total** (N=21)	5 (24%)	2.3 (1–4)	3.9 (3–5)	<0.01
IOP measurement (TonoPen)	PGY1 (N=7)	6 (86%)	3.3 (1–5)	4.75 (4–5)	0.01
	PGY2 (N=5)	5 (100%)	4.4 (3–5)	4.6 (4–5)	0.68
	PGY3 (N=9)	9 (100%)	4.6 (4–5)	4.9 (4–5)	0.17
	**Total** (N=21)	20 (95%)	4.1 (1–5)	4.8 (4–5)	0.01
Lateral canthotomy	PGY1 (N=7)	0 (0%)	1.3 (1–2)	3.1 (2–5)	<0.01
	PGY2 (N=5)	0 (0%)	1.6 (1–2)	3.8 (2–5)	<0.01
	PGY3 (N=9)	3 (33%)**	2.1 (1–4)	3.8 (2–5)	<0.01
	**Total** (N=21)	3 (14%)**	1.7 (1–4)	3.5 (2–5)	<0.01

IOP = intraocular pressure.

* Comfort level measured using Likert Scale (1–5): 1 = Not at all comfortable, 2 = Somewhat uncomfortable, 3 = Neutral, 4 = Somewhat comfortable, 5 = Very comfortable

** None had been performed independently but all in conjunction with ophthalmology.

**Figure:**
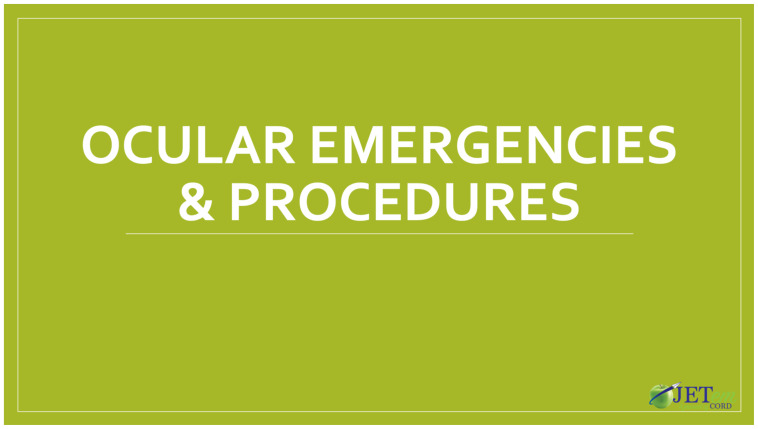
Please see associated PowerPoint file

## INNOVATION MATERIALS

### Appendix B OptimEYEzing Emergency Skills Pre-Survey

What is your current level of training (PGY-1, PGY-2, PGY-3, etc.)? _____________

#### Evaluation of Corneal Abrasion/Ulceration with fluorescein/slit lamp

Have you ever performed this procedure in clinical practice?

**Table:**

□ Yes	□ No

How comfortable do you feel performing this procedure?

**Table:**

Not at all comfortable	Somewhat uncomfortable	Neutral	Somewhat comfortable	Very comfortable
1	2	3	4	5

Have you ever consulted ophthalmology for assistance with this procedure?

**Table:**

□ Yes	□ No

#### Ocular Foreign Body Removal (needle tip removal)

Have you ever performed this procedure in clinical practice?

**Table:**

□ Yes	□ No

How comfortable do you feel performing this procedure?

**Table:**

Not at all comfortable	Somewhat uncomfortable	Neutral	Somewhat comfortable	Very comfortable
1	2	3	4	5

Have you ever consulted ophthalmology for assistance with this procedure?

**Table:**

□ Yes	□ No

#### Eye Pressure Measurement with TonoPen

Have you ever performed this procedure in clinical practice?

**Table:**

□ Yes	□ No

How comfortable do you feel performing this procedure?

**Table:**

Not at all comfortable	Somewhat uncomfortable	Neutral	Somewhat comfortable	Very comfortable
1	2	3	4	5

Have you ever consulted ophthalmology for assistance with this procedure?

**Table:**

□ Yes	□ No

#### Lateral Canthotomy

Have you ever performed this procedure in clinical practice?

**Table:**

□ Yes	□ No

How comfortable do you feel performing this procedure?

**Table:**

Not at all comfortable	Somewhat uncomfortable	Neutral	Somewhat comfortable	Very comfortable
1	2	3	4	5

Have you ever consulted ophthalmology for assistance with this procedure?

**Table:**

□ Yes	□ No

### Appendix C OptimEYEzing Emergency Skills Post-Survey

What is your current level of training (PGY-1, PGY-2, PGY-3, etc.)? _____________

#### Evaluation of Corneal Abrasion/Ulceration with fluorescein/slit lamp

After this course, how comfortable do you feel performing this procedure?

**Table:**

Not at all comfortable	Somewhat uncomfortable	Neutral	Somewhat comfortable	Very comfortable
1	2	3	4	5

In the future, would you plan to consult ophthalmology for assistance with this procedure?

**Table:**

□ Yes	□ No

#### Ocular Foreign Body Removal (needle tip removal)

After this course, how comfortable do you feel performing this procedure?

**Table:**

Not at all comfortable	Somewhat uncomfortable	Neutral	Somewhat comfortable	Very comfortable
1	2	3	4	5

In the future, would you plan to consult ophthalmology for assistance with this procedure?

**Table:**

□ Yes	□ No

#### Eye Pressure Measurement with TonoPen

After this course, how comfortable do you feel performing this procedure?

**Table:**

Not at all comfortable	Somewhat uncomfortable	Neutral	Somewhat comfortable	Very comfortable
1	2	3	4	5

In the future, would you plan to consult ophthalmology for assistance with this procedure?

**Table:**

□ Yes	□ No

#### Lateral Canthotomy

After this course, how comfortable do you feel performing this procedure?

**Table:**

Not at all comfortable	Somewhat uncomfortable	Neutral	Somewhat comfortable	Very comfortable
1	2	3	4	5

In the future, would you plan to consult ophthalmology for assistance with this procedure?

**Table:**

□ Yes	□ No

**Please include any feedback you have about the course below:**
