# Supplementary material for: OptimEYEzing Emergency Skills: A Novel Model for Ocular Procedural Education for Emergency Medicine Residents
Source: J Educ Teach Emerg Med. 2026 Apr 30;11(2):I21–33. doi: 10.5070/M5.52212 (PMC13152363; doi:10.5070/M5.52212)
Supplement: Supplementary file 1 [file 11-2-I21-Appendix_A.pptx]

## Slide 1
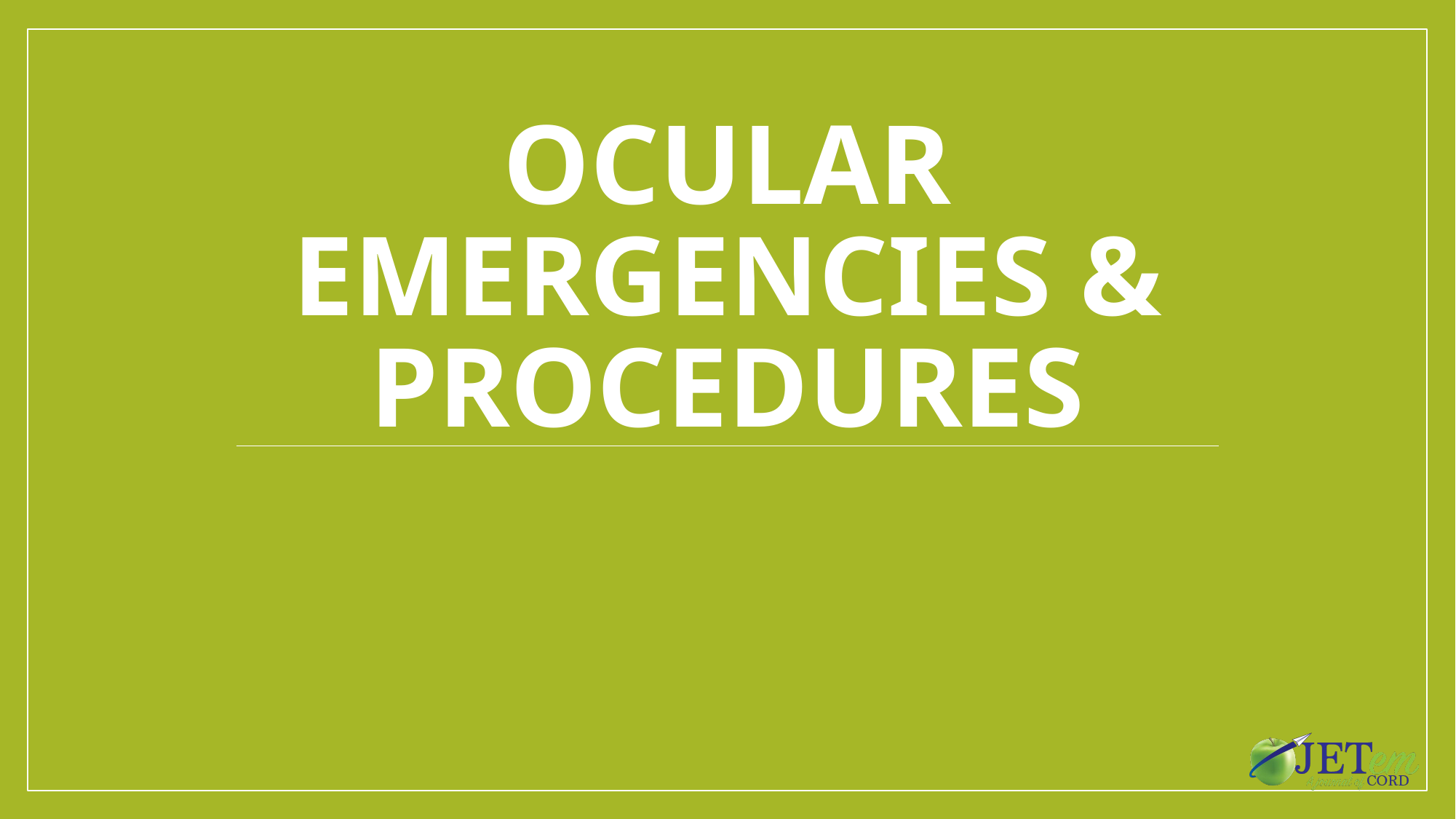

# Ocular Emergencies & Procedures

## Slide 2
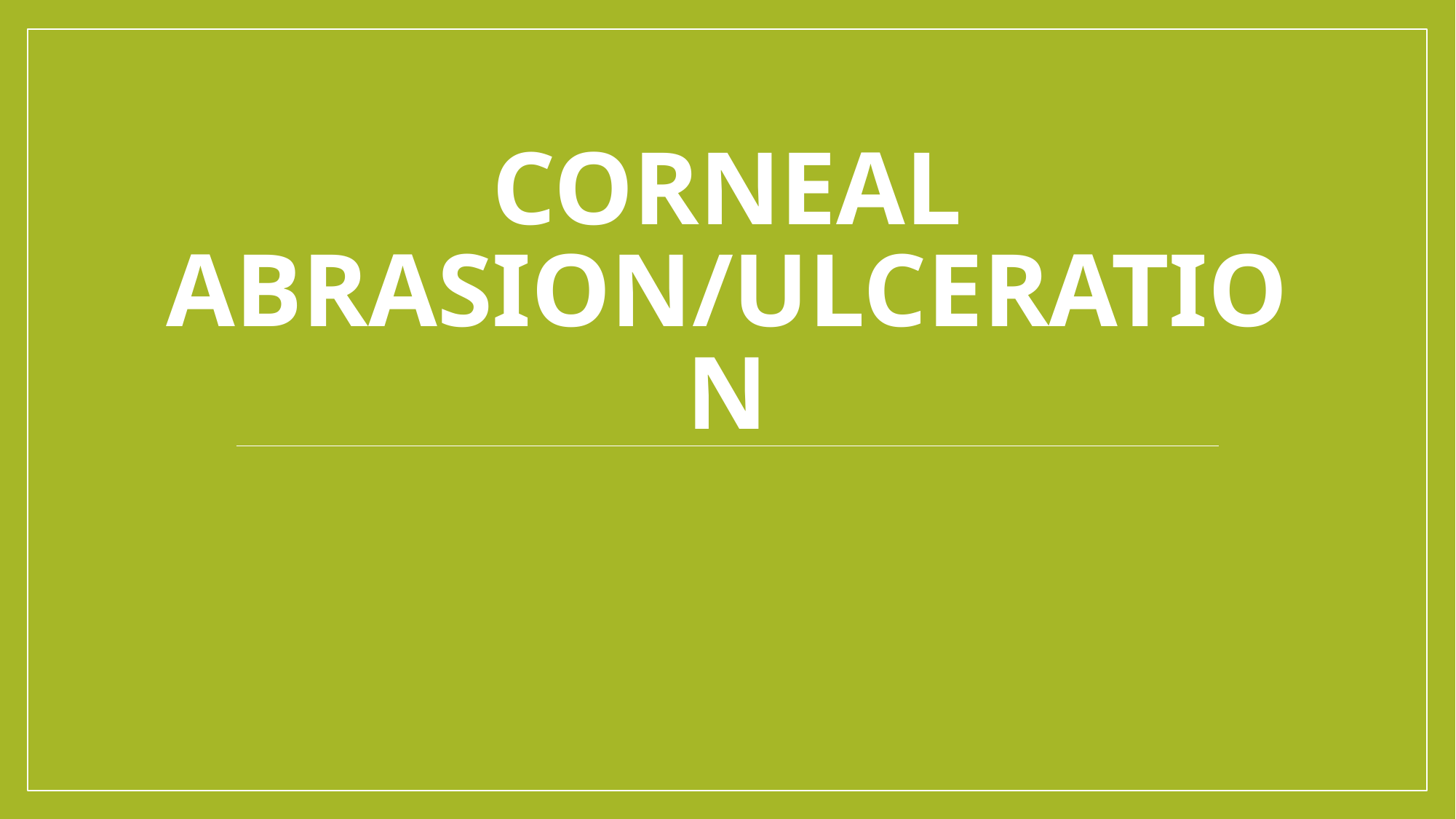

# Corneal Abrasion/Ulceration

## Slide 3
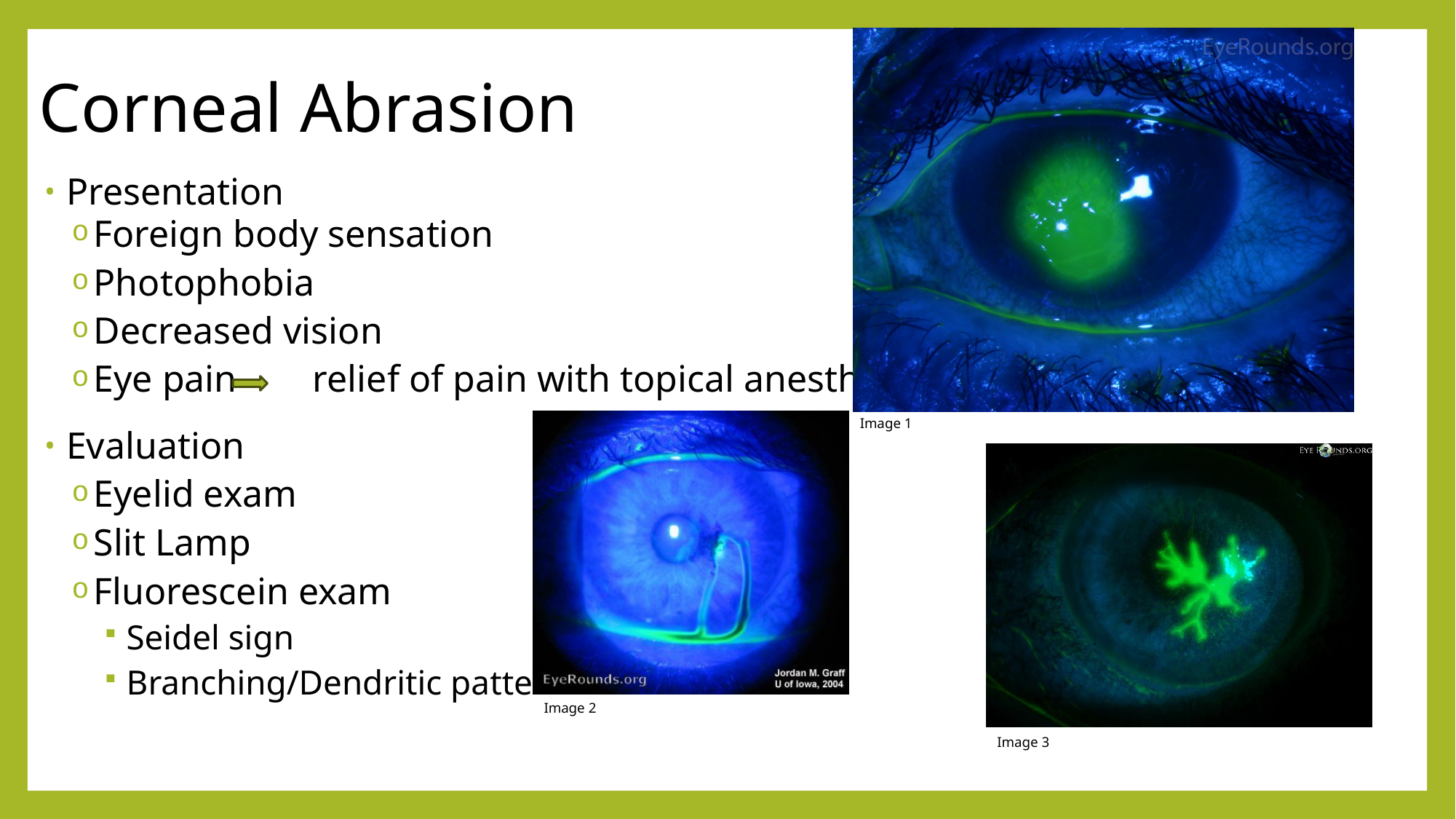

# Corneal Abrasion
Presentation
Foreign body sensation
Photophobia
Decreased vision
Eye pain        relief of pain with topical anesthesia
Evaluation
Eyelid exam
Slit Lamp
Fluorescein exam
Seidel sign
Branching/Dendritic pattern
Image 1
Image 2
Image 3

## Slide 4
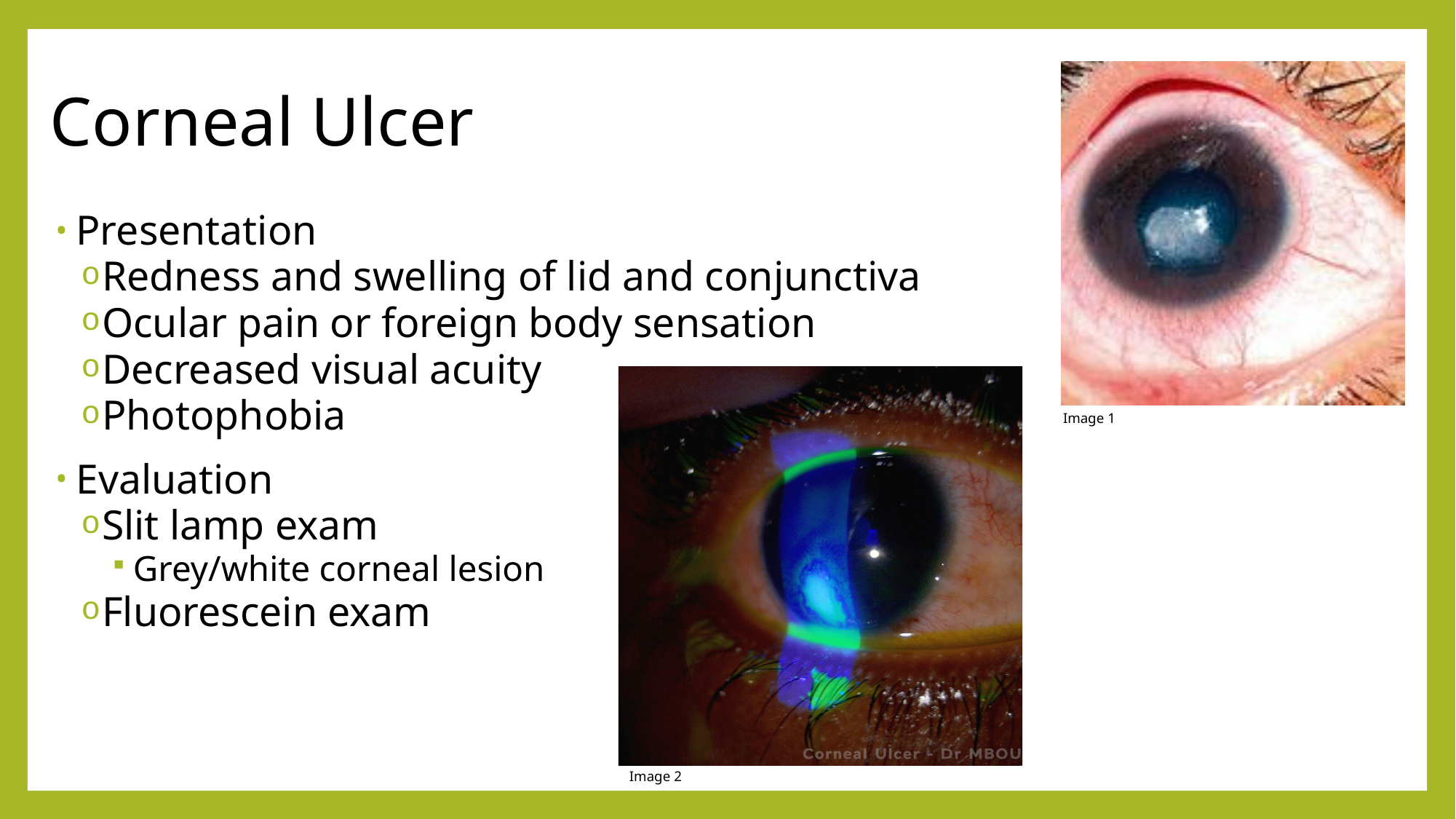

# Corneal Ulcer
Presentation
Redness and swelling of lid and conjunctiva
Ocular pain or foreign body sensation
Decreased visual acuity
Photophobia
Evaluation
Slit lamp exam
Grey/white corneal lesion
Fluorescein exam
Image 1
Image 2

## Slide 5
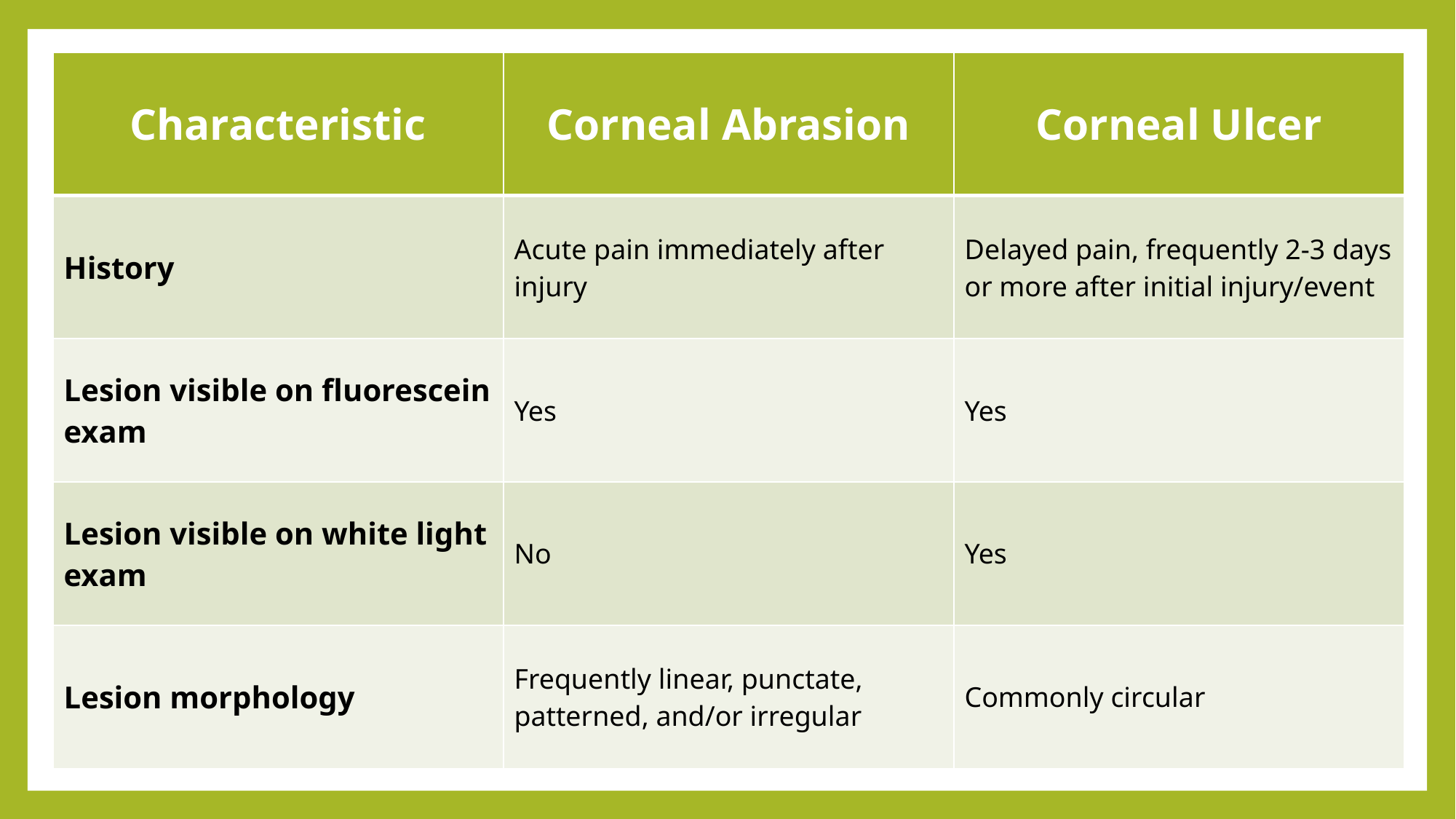

| Characteristic | Corneal Abrasion | Corneal Ulcer |
| --- | --- | --- |
| History | Acute pain immediately after injury | Delayed pain, frequently 2-3 days or more after initial injury/event |
| Lesion visible on fluorescein exam | Yes | Yes |
| Lesion visible on white light exam | No | Yes |
| Lesion morphology | Frequently linear, punctate, patterned, and/or irregular | Commonly circular |

## Slide 6
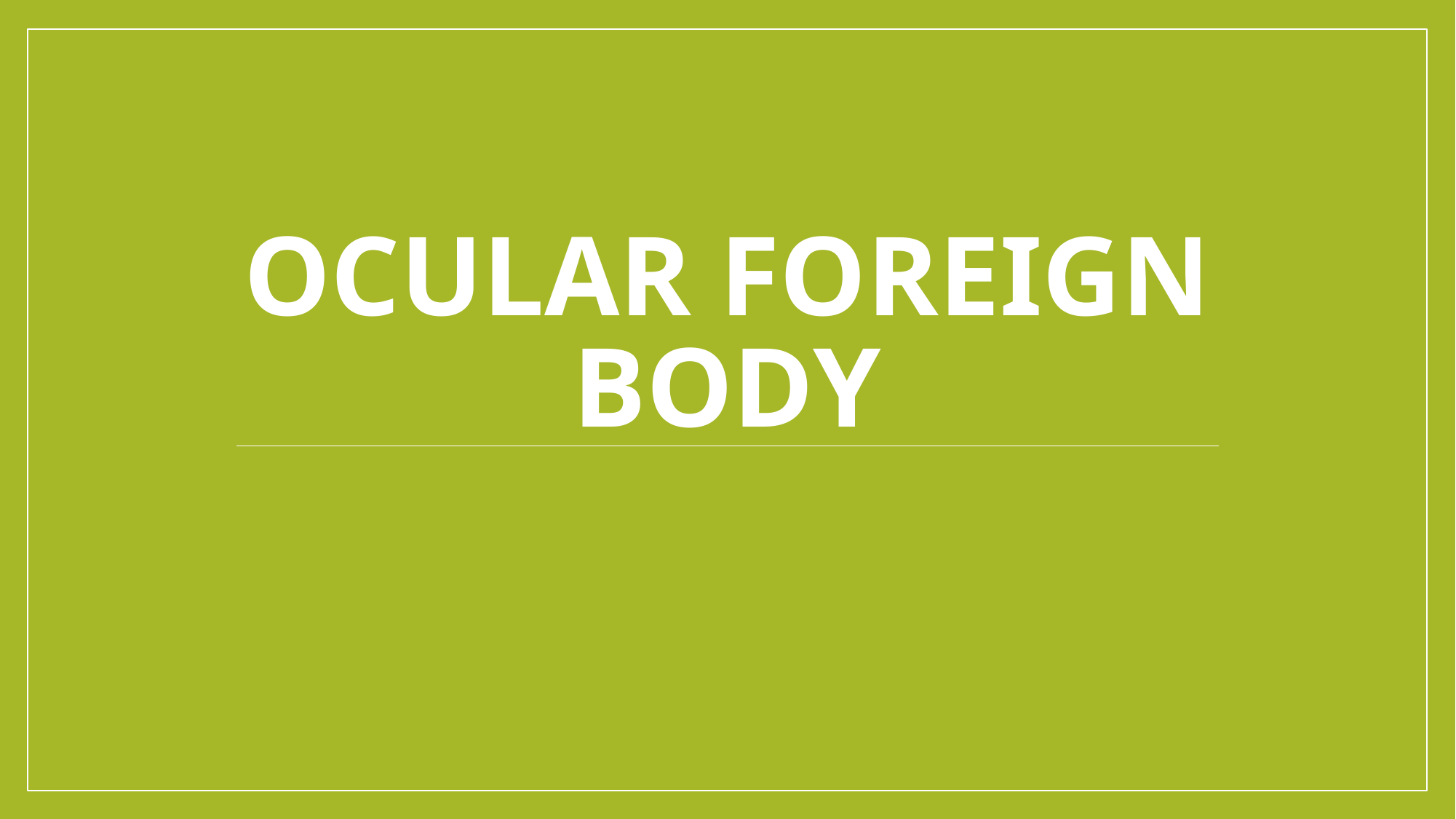

# Ocular Foreign Body

## Slide 7
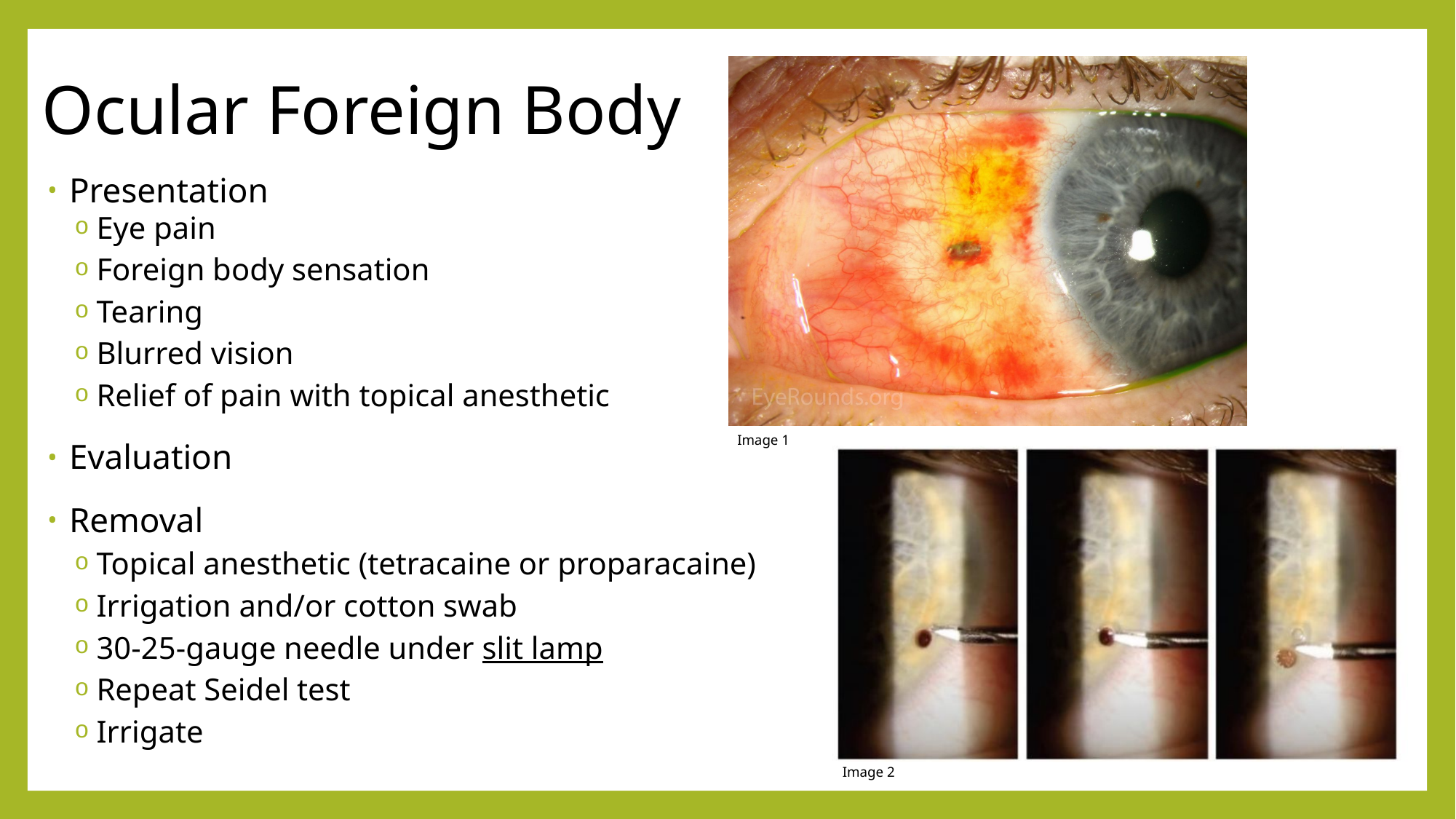

# Ocular Foreign Body
Presentation
Eye pain
Foreign body sensation
Tearing
Blurred vision
Relief of pain with topical anesthetic
Evaluation
Removal
Topical anesthetic (tetracaine or proparacaine)
Irrigation and/or cotton swab
30-25-gauge needle under slit lamp
Repeat Seidel test
Irrigate
Image 1
Image 2

## Slide 8
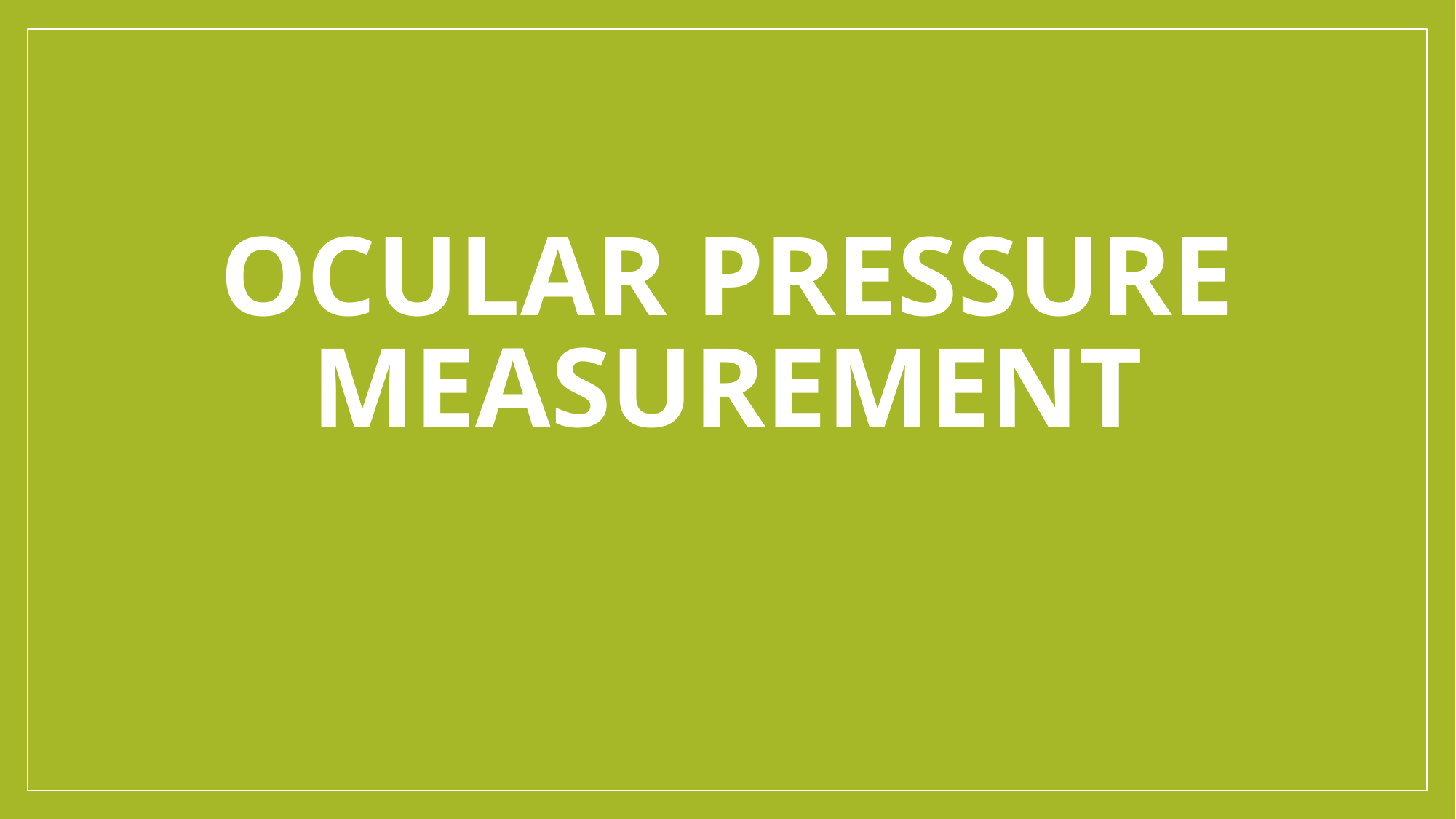

# OCular Pressure Measurement

## Slide 9
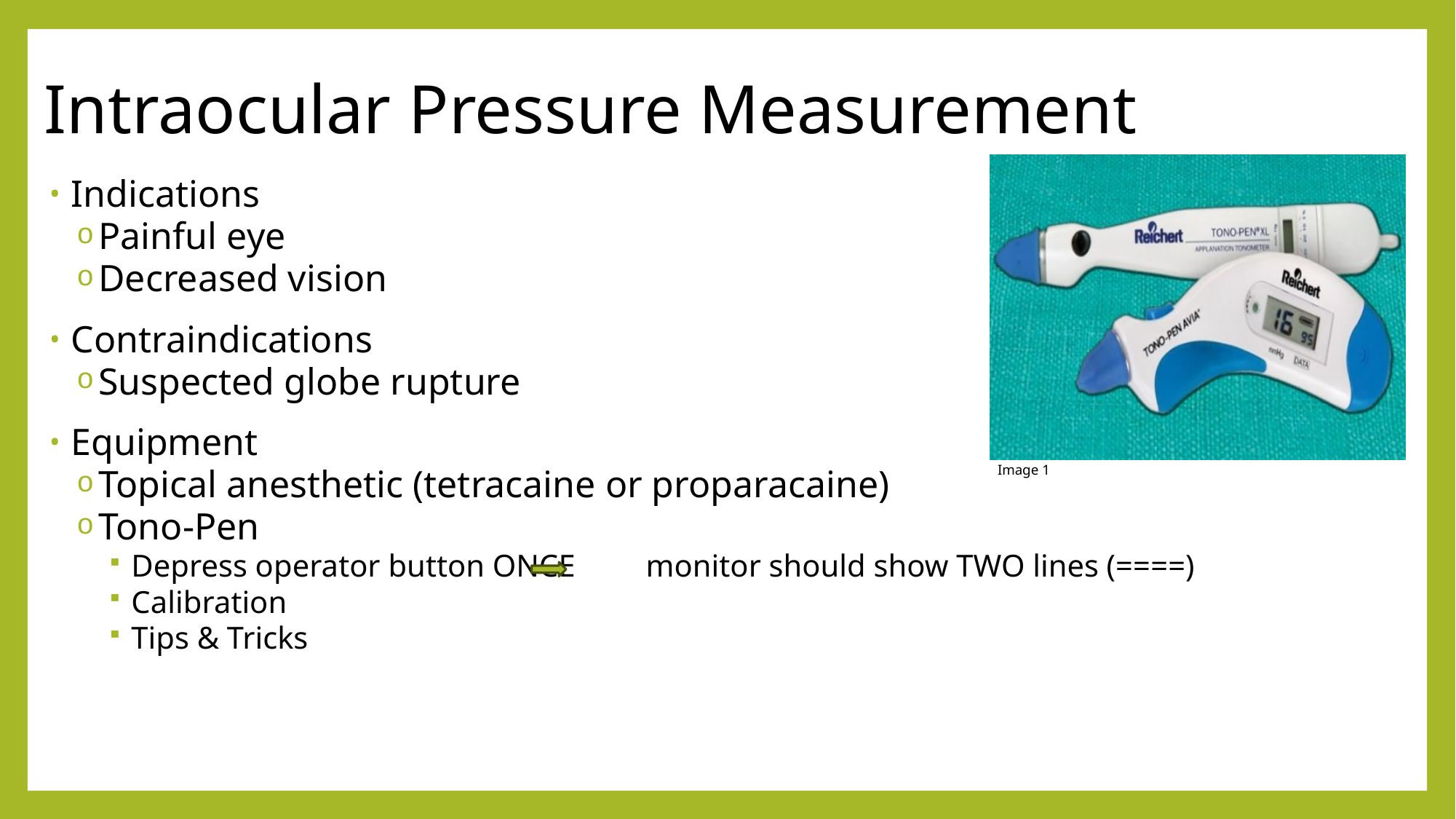

# Intraocular Pressure Measurement
Indications
Painful eye
Decreased vision
Contraindications
Suspected globe rupture
Equipment
Topical anesthetic (tetracaine or proparacaine)
Tono-Pen
Depress operator button ONCE         monitor should show TWO lines (====)
Calibration
Tips & Tricks
Image 1

## Slide 10
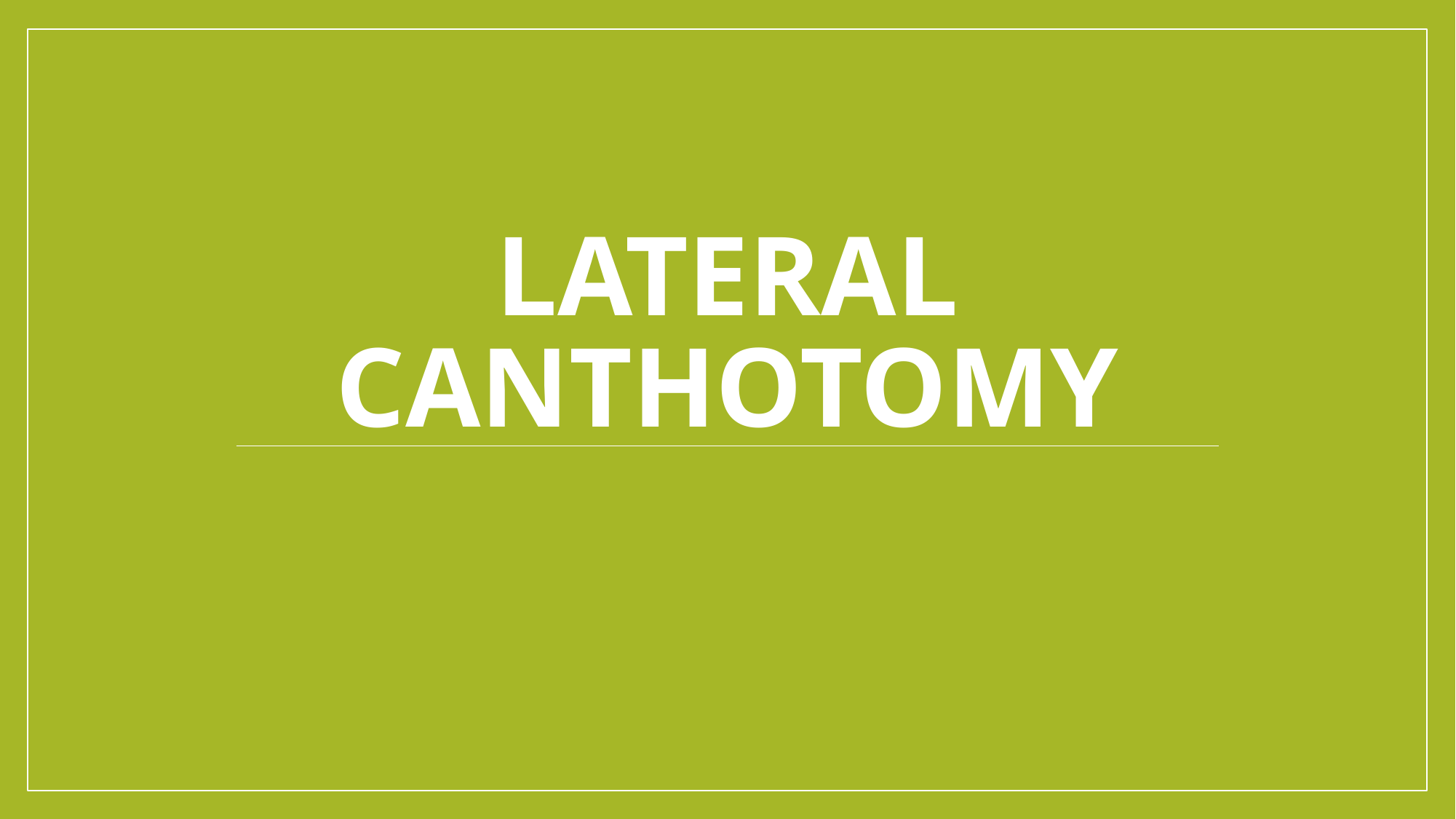

# Lateral Canthotomy

## Slide 11
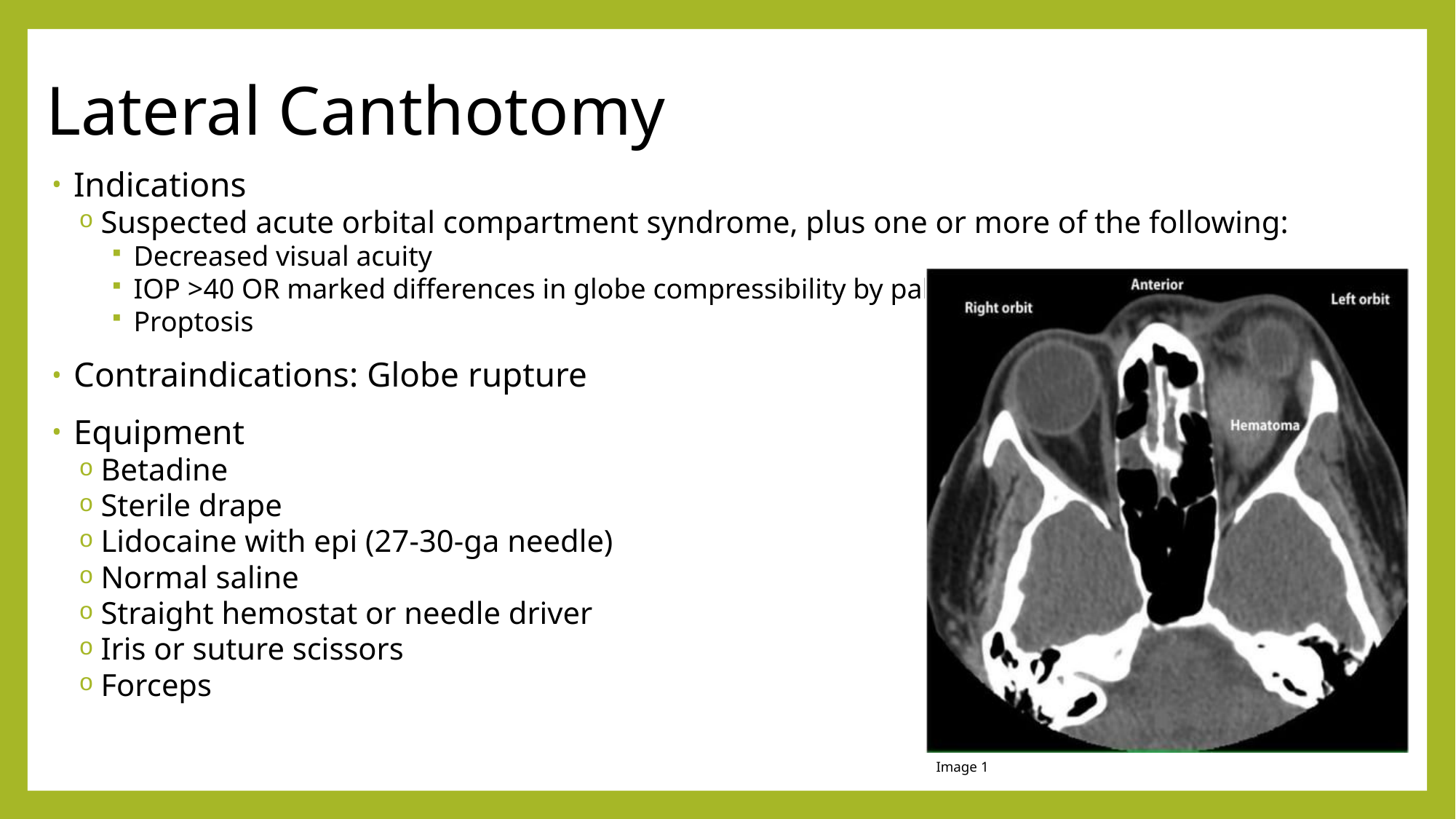

# Lateral Canthotomy
Indications
Suspected acute orbital compartment syndrome, plus one or more of the following:
Decreased visual acuity
IOP >40 OR marked differences in globe compressibility by palpation
Proptosis
Contraindications: Globe rupture
Equipment
Betadine
Sterile drape
Lidocaine with epi (27-30-ga needle)
Normal saline
Straight hemostat or needle driver
Iris or suture scissors
Forceps
Image 1

## Slide 12
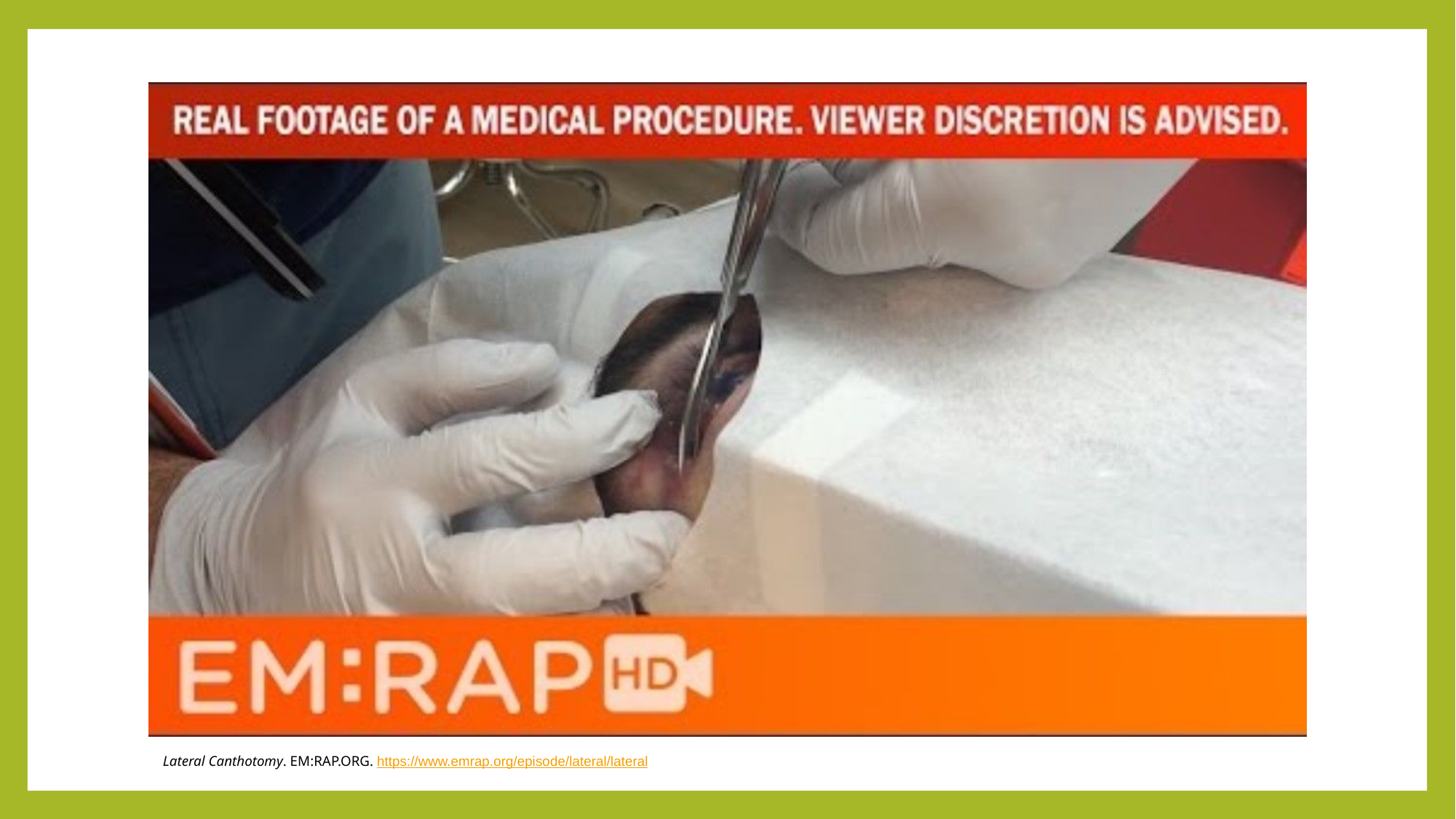

Lateral Canthotomy. EM:RAP.ORG. https://www.emrap.org/episode/lateral/lateral

## Slide 13
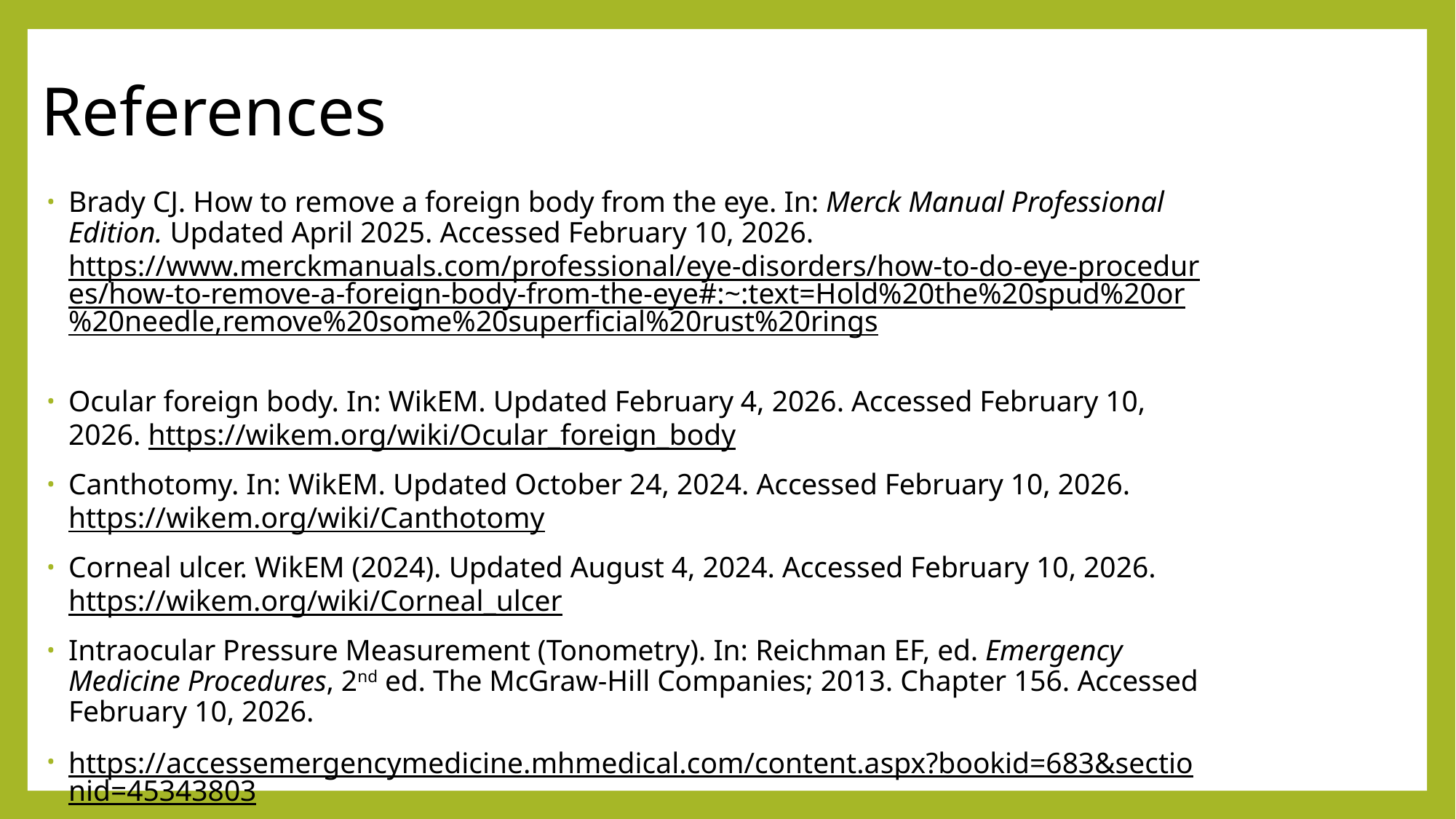

# References
Brady CJ. How to remove a foreign body from the eye. In: Merck Manual Professional Edition. Updated April 2025. Accessed February 10, 2026. https://www.merckmanuals.com/professional/eye-disorders/how-to-do-eye-procedures/how-to-remove-a-foreign-body-from-the-eye#:~:text=Hold%20the%20spud%20or%20needle,remove%20some%20superficial%20rust%20rings
Ocular foreign body. In: WikEM. Updated February 4, 2026. Accessed February 10, 2026. https://wikem.org/wiki/Ocular_foreign_body
Canthotomy. In: WikEM. Updated October 24, 2024. Accessed February 10, 2026. https://wikem.org/wiki/Canthotomy
Corneal ulcer. WikEM (2024). Updated August 4, 2024. Accessed February 10, 2026. https://wikem.org/wiki/Corneal_ulcer
Intraocular Pressure Measurement (Tonometry). In: Reichman EF, ed. Emergency Medicine Procedures, 2nd ed. The McGraw-Hill Companies; 2013. Chapter 156. Accessed February 10, 2026.
https://accessemergencymedicine.mhmedical.com/content.aspx?bookid=683&sectionid=45343803
